# Convergent Evidence of Eagle Talons Used by Late Neanderthals in Europe: A Further Assessment on Symbolism

**DOI:** 10.1371/journal.pone.0101278

**Published:** 2014-07-10

**Authors:** Matteo Romandini, Marco Peresani, Véronique Laroulandie, Laure Metz, Andreas Pastoors, Manuel Vaquero, Ludovic Slimak

**Affiliations:** 1 Università degli Studi di Ferrara, Dipartimento di Studi Umanistici, Sezione di Scienze Preistoriche e Antropologiche, Corso Ercole I d’Este 32, Ferrara, Italy; 2 CNRS, PACEA UMR5199, Université de Bordeaux, Talence, France; 3 UMR 7269 LAMPEA, Université Aix-Marseille, Maison Méditerranéenne des Sciences de l’Homme, Aix-en-Provence, France; 4 Neanderthal Museum, Mettmann, Germany; 5 Institut Català de Paleoecologia Humana i Evolució Social (IPHES), Zona Educacional 4 - Campus Sescelades URV, Tarragona, Spain; 6 CNRS, UMR 5608, Université de Toulouse 2 le Mirail, Maison de la Recherche Bât 26, Toulouse, France; University of Oxford, United Kingdom

## Abstract

To contribute to have a better understanding of the symbolic or not use of certain items by Neanderthals, this work presents new evidence of the deliberate removal of raptor claws occurred in Mediterranean Europe during the recent phases of the Mousterian. Rio Secco Cave in the north-east of Italy and Mandrin Cave in the Middle Rhône valley have recently produced two golden eagle pedal phalanges from contexts not younger than 49.1–48.0 ky cal BP at Rio Secco and dated around 50.0 ky cal BP at Mandrin. The bones show cut-marks located on the proximal end ascribable to the cutting of the tendons and the incision of the cortical organic tissues. Also supported by an experimental removal of large raptor claws, our reconstruction explains that the deliberate detachment occurred without damaging the claw, in a way comparable at a general level with other Mousterian contexts across Europe. After excluding that these specimens met the nutritional requirements for human subsistence, we discuss the possible implications these findings perform in our current knowledge of the European Middle Palaeolithic context.

## Introduction

**Figure 1:**
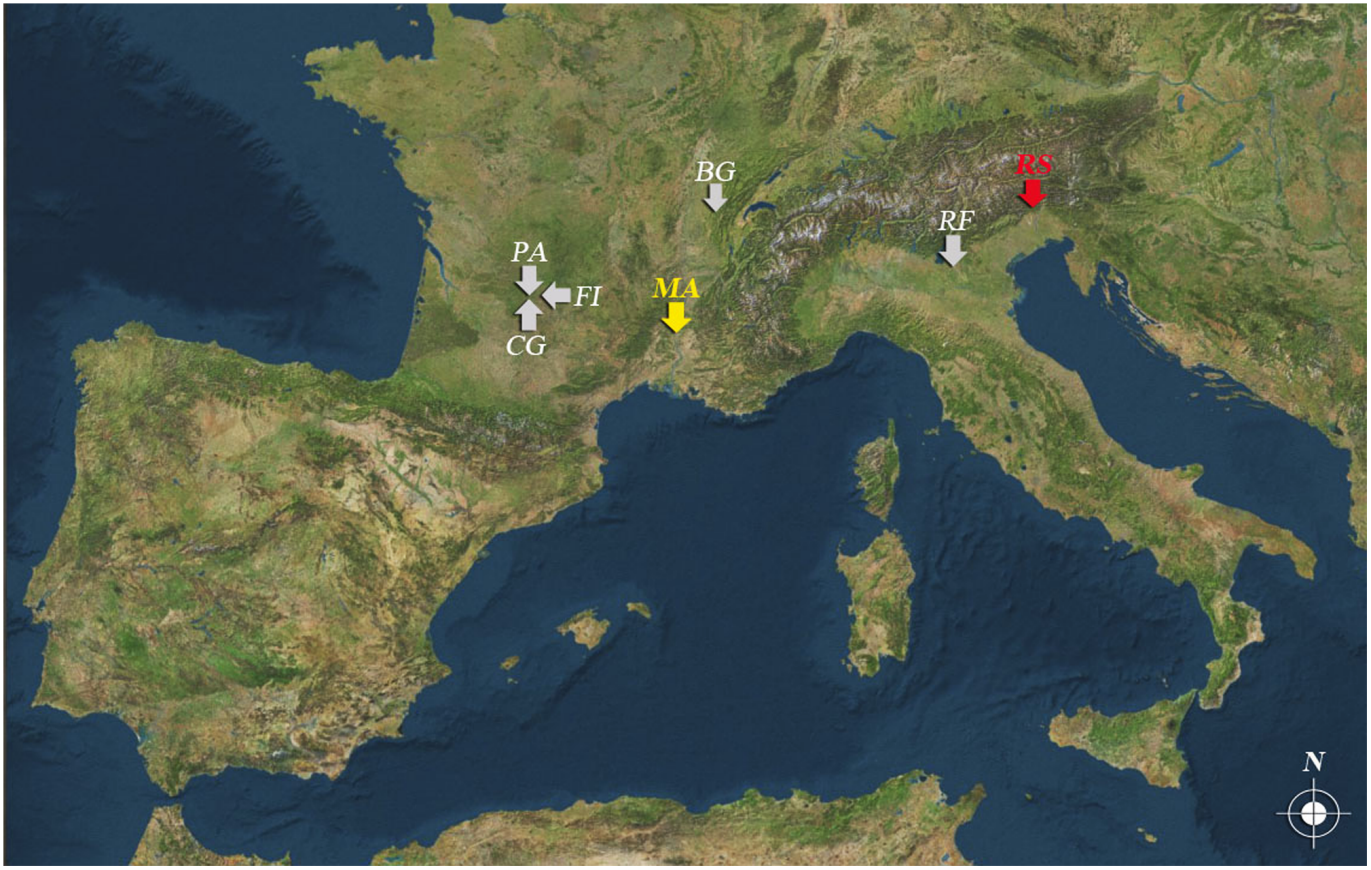
Position of the Middle Palaeolithic sites with cut-marked raptor phalanges in Europe. CG, Combe Grenal; FI, Le Fieux; PA, Pech de l’Azé; BG, Baume de Gigny (swan); MA, Mandrin; RF, Fumane; RS, Rio Secco.

Evidence of interactions between large raptors and hominins is very scanty if not ephemeral. The correlation between Middle Palaeolithic sites and the incidence of raptors and corvid bones, especially wing bones, recorded across a vast area from Europe to the Levant suggest that Neanderthals may have exploited black feathered birds in a number of sites [1]. However, direct evidence of such exploitation remains exceptional and the few taphonomic analyses undertaken lead us to caution [2]. In some cases, alimentary exploitation has been excluded due to evidence of specific selection of plumage and talons, clearly expressed by the presence of precisely localized cutmarks to remove the middle-distal wing with grafted remix feathers, or the claw. Evidence has been produced from Fumane cave in Italy, where the avifaunal sample shows cutmarks on bones of medium (red-footed falcon *Falco vespertinus*) and large-sized raptors (lammergeier *Gypaetus barbatus*, Eurasian black vulture *Aegypius monachus*) in association with other birds (Alpine chough *Pyrrhocorax graculus* and Common wood pigeon *Columba palumbus*) processed for the same purpose [3], suggesting a symbolic use of particular parts of the bird body. Besides wings and related feathers, another particular aspect of the use of bird elements concerns the removal of the claw. Isolated pedal phalanges of golden eagle (*Aquila chrysaetos*), white-tailed eagle (*Haliaeetus albicilla*), Cinereous Vulture (*Aegypius monachus*), an indeterminate medium-size raptor, as well as swan (*Cygnus cygnus*) have been found in France at Pech de l’Azé I [4]–[5], Baume de Gigny [6], Pech de l’Azé IV [7]–[8], Combe-Grenal and Les Fieux [9] and, again, at Grotta di Fumane [10], in different periods, from about 100 ky BP to 44–45 kyBP, revealing a certain convergence of such selections (Figure 1). These posterior phalanges bear disarticulation striae showing the successful removal of the claw from the toe using lithic tools. Traces ascribable to the removal of pedal phalanges of diurnal and nocturnal raptors have also been observed from sites across the Upper Palaeolithic in Europe and the Near-east, like La Quina [11], Gönnersdorf and Andernach [12], Meged Rockshelter [13], Üçağızlı Cave [14], Bois-Ragot [15]–[16] or le Morin [17]. Because claws are not directly compatible with human consumption, they are derived from activities unrelated to food processing and may have served in a limited range of actions, likely symbolically oriented [9]. An attractive hypothesis is an ornamental use due to their length and curvature, and possibly their suspension in different ways, a common feature for such elements in the ethnographic records. This interpretation of pedal phalanges’ use as pendants, suspended in isolation or still connected to the entire foot has also been supported from the over-representation of raptor feet bones recorded for more recent periods of the Upper Palaeolithic at Meged [13] and Ohalo II [18].

Concerning Neanderthal populations for whom no symbolically mediated behaviours have been unambiguously demonstrated, such traits are proposed on the existence of actions that appear to the archaeologist as indirectly utilitarian and/or invested of sub-rational traits, as expressed in their relation with death through Neanderthal graves and specific forms of cannibalism [19]–[22]. Specifically, Valensi et al. [22] reveal higher percentage of cut marks on the human remains (over 50%) by comparison to the deer bones (11%), which implies that Neanderthals from Moula-Guercy, layer XV, took great care in cutting up the bodies. Neandertals were here eaten, but these actions took place in a particularly different relation than with any other mammals.

Neanderthal symbolically mediated behaviours have also been recently proposed by the discovery of shells interpreted as ornamental objects. One must underline that shells present a natural hole, unrelated to any anthropic modification or use, but traces of colouring materials suggest that these may be interpreted like ochered pendants [23] or as colorant containers [24]. Red and black pigments derived from the pulverization of manganese oxide or hematite is clearly attested during the late Mousterian and is hypothesized to have been used on soft materials, like human skin to create corporeal designs, but their true function(s) remain unknown [25].

Aside from the rare pebbles and stone artifacts that appear to be lined by various types of incisions, either intentional or of secondary origin, and which are subject to various interpretive uncertainties [26], shells, as well as raptor claws, remain the best evidence to suggest symbolic expression by Neanderthal populations previous to the arrival of *Homo sapiens* in Europe. A very controversial issue concerns the transition between the Middle and Upper Palaeolithic and the Castelperronian culture, where pendants and bone tools, attested at Quinçay [27] but mainly at the Arcy-sur-Cure site, are interpreted in opposing ways. These are seen as either stratigraphic pollution from the overlying Upper Palaeolithic layers (e.g. [28]–[29]) or as belonging entirely to Castelperronian activities (e.g. [30]–[31]), while others warn on the uncertain attribution of the Castelperronian to Neanderthal populations [32]. Such divergent results underline that, some 60 years after the end of the excavations of these archaeological layers, any scientific demonstration of Neanderthal symbolism in Arcy remains disputable. One must keep in mind that in the current archaeological records of the Middle Palaeolithic of Europe even the most simple design creations (e.g. geometric engravings, basic paintings, or simply the manufacturing of a hole for the suspension of an object) that would provide positive evidence of Neanderthal symbolically mediated behaviour remain unambiguously absent (see [26] for a discussion on disputable cases). It is noteworthy that such positive evidence is only attested through Eurasia by a tenth of thousands of cases for all Upper Palaeolithic, revealing a shared behavioural trait of any *Homo sapiens* societies. Such lack of positive evidence is a particularly suggestive trait of the Neanderthalian populations, and research on the existence of symbolic features can therefore be built exclusively out of negative evidence, through the collect of structurally unmodified elements, like wings and feathers, fossils and shells. These have, nevertheless, the potential to be interpreted as pendants and, as such, belong to a codified mediation between individuals.

In our current knowledge of Neanderthal societies, even negative evidence remains an exceptional find and is attested by less than a tenth of cases. In order to have a better understanding of these scanty records, we present new evidence for the deliberate removal of raptor claws produced from two different sites, in the Italian pre-Alps and in Mediterranean France, from the recent phases of the Mousterian. An experimental removal of large raptor claws has been conducted for a better understanding of the striae formation on the bones, the gestures performed, and the dynamics of this specific type of interaction.

## Results

### Experiencing the deliberate removal of large raptor claws

**Figure 2:**
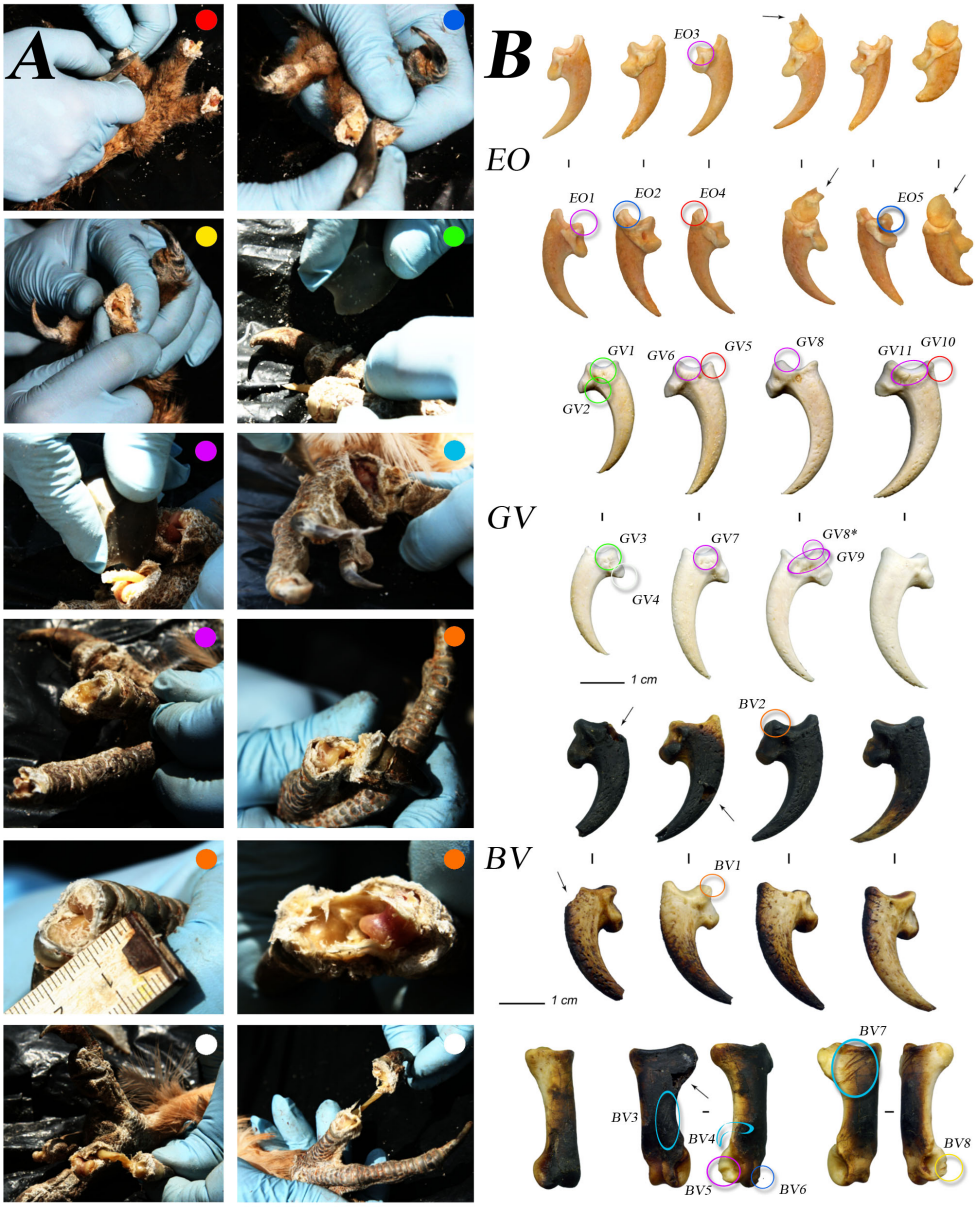
Experimental butchering of claws. A) different stages of the experimental extraction of large raptors claws: red, dorsal disarticulation; blue, plantar dis.; yellow, manual dis. due to bending opposite to the articulation; green, lateral dis.; violet, cut and exposure of the lateral ligaments; light blue, proximal phalanges dis.; orange, manual dis. due to bending following the articulation and exposure of the lateral ligaments; white, extraction of the claws and exposure of the *tendon flexor profound*. B) experimental bony core of claws with localization of the traces imprinted and references of the close-ups in Figure 5, the colors are related to the different experimental phases (A). The arrows indicate the *arrachement* marks caused by the manual disarticulation. EO = Eagle-Owl (*Bubo bubo*); GV = Griffon Vulture (*Gyps fulvus*); BV = Bearded Vulture (*Gypaetus barbatus*).

An experimental study was carried out to remove all of the ungual phalanges (Figure 2, A–B) and keratin of prime examples of an adult male bearded vulture (*Gypaetus barbatus*), a griffon vulture (*Gyps fulvus*), and an eagle-owl (*Bubo bubo)*. This follows a first experiment of disarticulation, when striae as epiphenomena were noticed on 7 snowy owl claws [15], cut with an unretouched tool.

Disarticulation and incising of the digits were done using blades and retouched Levallois and Discoid points. The first were not suitable to cut into the thick skin, but were effective at severing the strong ligaments and tendons; the second, thanks to the retouched margin, was more effective in sawing operations to cut the stratum corneum, so as to expose the joints.

The experiment exposed at least three types of traces around the most extreme proximal articular surfaces, based on their location: dorsal, lateral/medial ventral, and ventral. Their formation is strictly dependent on the mode with which they were detached from the claw. The choice lies on the opening of the tissues into either a dorsal or plantar position (Figure 2, red-blue), as it was extremely inconvenient and time-consuming to proceed from the medial and lateral sides (Figure 2, green). The first case cited is transversally incised by sawing or single, decisive gestures at the portion confined between the proximal end of the keratin; the second case, between the plantar fingertip and the articulation with the penultimate phalanx and the same talon. In both situations, the manual flexion (Figure 2, yellow) of the articulation (in favor or opposed) was facilitated by the cuts inflicted. In the course of the opposed gesture, partial damage to the cortical bone tissue on the two articular epiphyses occurred, comparable to *arrachement* marks [33]. The same flexion, if conducted without prior incision of the tissue, can also cause fracturing (depending on the finger), corresponding with the distal shaft of the digital phalanx, before the articulation with the bony claw (Figure 2, A). Operating instead with a lithic tool on the dorsal surface, it was observed that exposure of the joint facilitates the severing of the cruciate and lateral ligaments (Figure 2, orange), as it affected the proximal part of the claw keratin. From the plantar view, the tool cut the *flexor profundus* tendon, which affected the bony core of the claw, leaving the lateral ligament intact, to be cut in the second phase. Dorsally, the *flexor profundus* tendon (normally 10 cm long) is spared and removed along with the whole claw following the lateral release of the joint (Figure 2, white).

In completion, the operation of extraction/disarticulation of the claws was difficult and not immediate (normally 3–4 minutes). The tools and the modalities, however, yield effective results. Once cleaned, the experimentally cut claws display a wide range of traces (Figure 2, BV + indicator), although in rare cases these are not registered. Some elements have been deliberately thermally altered to verify the resistance of the cut-marks to this type of agent.

**Figure 3:**
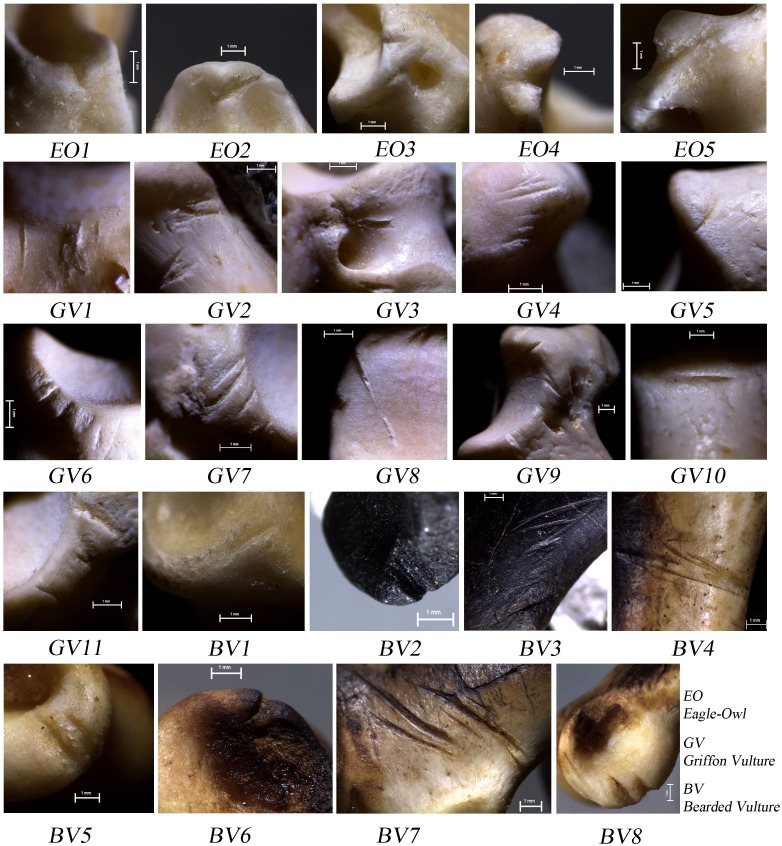
Close-ups of the traces in figure 2. EO = Eagle-Owl; GV = Griffon Vulture; BV = Bearded Vulture.

**Figure 4:**
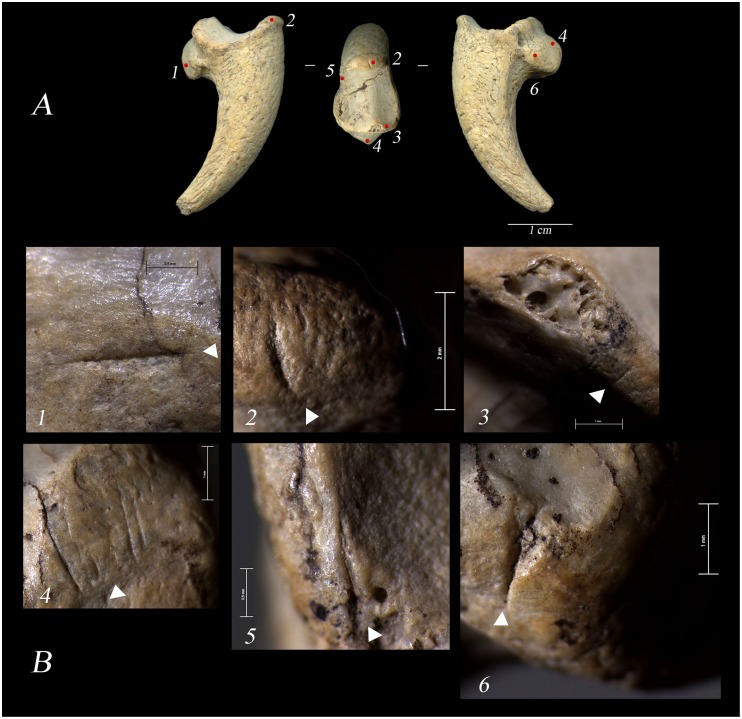
The cut-marked bone from Rio Secco. A) complete terminal pedal phalanx of a cfr. Golden Eagle (*Aquila* cfr. *chrysaetos*), n° RIL. 188. The numbers, 1–6, indicate the localization of the recognized anthropic traces. B) close-up of the traces oriented according to figure A.

**Figure 5:**
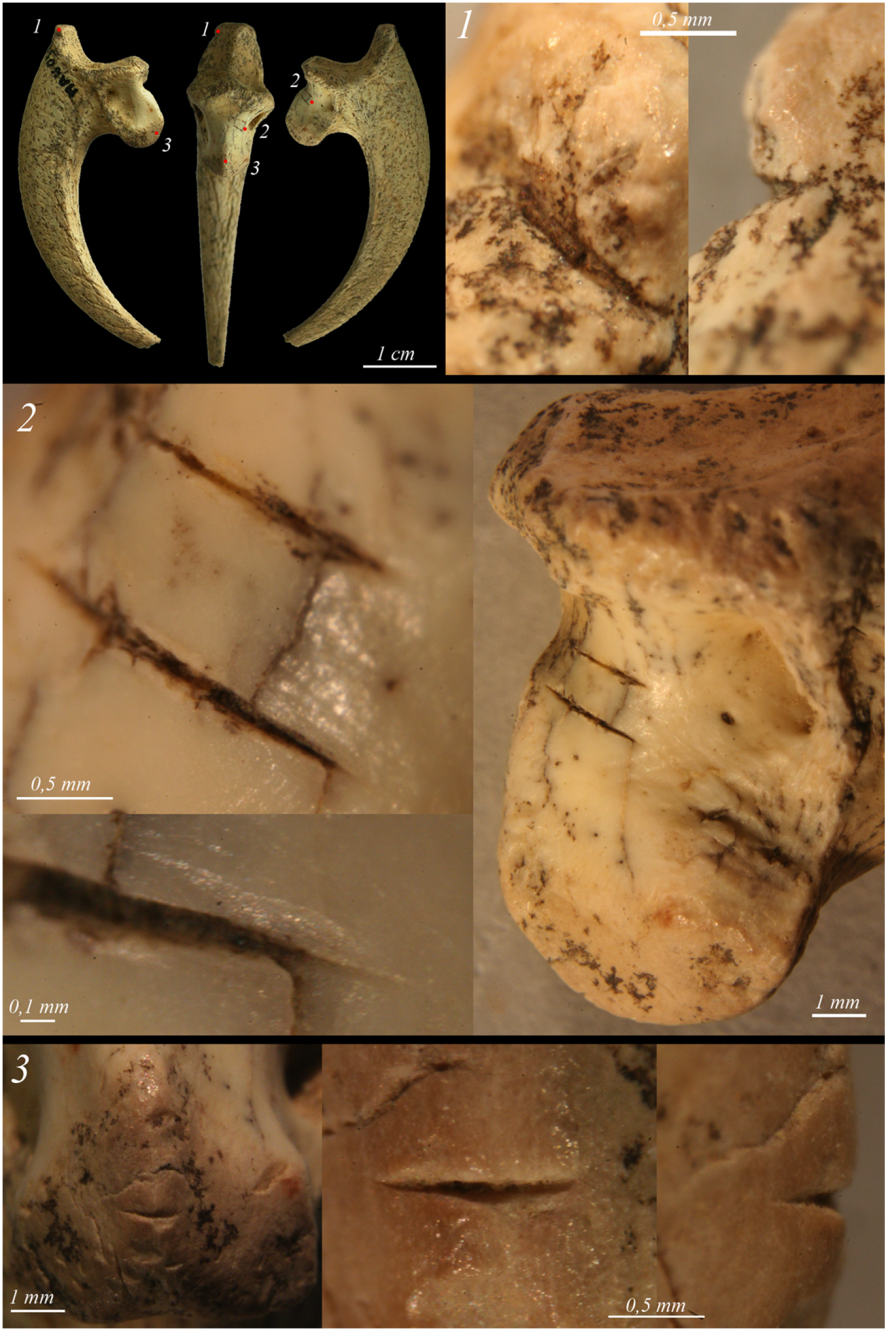
The cut-marked bone from Mandrin cave. Mandrin (n° MAN05 E 474), talon of Golden Eagle *Aquila chrysaetos*, showing cut-marks (photo VL).

Of the fifteen experimental bony claws analyzed, only three are not notched by a lithic edge. Where the lithic tool acted dorsally, the tracks (like Les Fieux and Combe Grenal) are repeated constantly (Figure 2, EO4-GV5-GV10). Starting from the back, once the joint has been exposed, detaching the element must necessarily sever the *flexor profound* tendon, producing clearly distinguishable traces (Figure 2_BV2–BV1). From the opposite point of view, the gesture seems to affect the upper/dorsal portion of the articular facet and the base of the bony claw (Figure 2, EO2-EO5-BV6-BV8; like Fumane, Rio Secco and Mandrin). Beginning the disarticulation laterally (Figure 2, GV1 through GV3) confirms the short and deep cut-marks already highlighted during the experimental removal. In all three cases, acting on the resistant lateral and medial ligaments produced the greatest number of traces (Figure 2, EO1-GV6 through GV9-GV11-BV5). Striae on the phalanx digit like at Pech de l’Azé I, level 4, occur both during the disarticulation of the claw and the entire digit from the tarsometatarsus (Figure 2, light blue; Figure 3, BV3-BV4-BV6-BV7). Finally, only with determined removal or damage to the *flexor profundus* tendon at the base of the pedal phalanx can striae perpendicular to the major axis (Figure 2, GV4) of the element be created (like Rio Secco and Mandrin, Figure 4, 4, Figure 5, 3).

In the bony claws, the *arrachement* marks caused by the manual disarticulation by opposing flexion have not been produced, but in the case of the eagle-owl, the more distal digital phalanx endured the fracture of the diaphysis up to the neck of the distal articulation (Figure 2, EO+indicator). In the course of this operation, because of the force used, a bending fracture, which has compromised the original form of the keratin case, was also produced.

### Rio Secco cave

Rio Secco cave is situated in a stream gorge at 580 m asl on the Pradis Plateau in the eastern part of the Carnic Pre-Alps, an orographic system dissected from N–S and W–E oriented valleys that separate mountains with peaks of 2,000–2,300 m asl. The Pradis Plateau comprises an area of 6 sq kms, enclosed on three sides by mountains peaking from 1,148 m to 1,369 m and to the south by the foothills, facing the present-day Friulian alluvial plain flooded from the Tagliamento River and its tributaries. The site is a flat and wide shelter facing south, with a gallery completely filled with sediments, which in the outer area of the shelter are limited to a heap of large boulders suggesting that the original roof was more vast than in present-day. Discovered in 2002 and systematically investigated since 2010, the cavity contains a sedimentary succession made of stones and loams that records human occupations during the late Middle Palaeolithic and the mid Upper Palaeolithic [34]–[35]. The late Mousterian sequence is recorded in layers 5top, 7, 5 and 8, a set of sedimentary units grouped in the macro-unit labeled BIO1 due to the intense bioturbation caused by the activity of burrowing animals (marmots, *Marmota marmota*), who are responsible for mixing fine fraction, displacing portions of anthropic sediment, and locally scattering flint implements, bones and charcoals. At the top there is a brown horizon (layer 5top), with stones and loamy fine fraction with portions still in place, or slightly deformed, and dark loamy horizons with organic material, bones and lithic implements (parts of layers 7 and 8) (Figure S1). Marmot and other animal dens and tunnels are filled up with sediments of different color, porosity and consistency. The net of tunnels is denser at the entrance than in the inner cavity. Layer 7 is comprised of loamy, dark yellowish-brown sediment with stones found under the cave vault. It is absent in the external zone, due to burrowing activity. The upper boundary with layer 5top is marked by an increasing frequency of bones and lithics, some of which bear also signatures of accidental heating. Layer 5 is a stony level of variable thickness with dispersed bones and flint implements between layer 7 and the thick anthropogenic layer 8, which is loamy with abundant stones, and contains tiny charcoals, small burned and unburned bones and several lithic implements. The lithic artifacts comprise tiny scrapers and flakes made of flint.

Chronometry of layers 7 and 8 shows that a lower calibrated boundary cannot be older than 49,000 14C BP, whereas the upper boundary of layer 7 ranges from 49,120 to 47,940 cal BP [36].

The claw of the cfr. *Aquila chrysaetos* (golden eagle) was identified and recorded (RIL188; Q.I15II) during the excavation campaign of 2013, on the wall of a marmot tunnel (Figure S1, B–D) dug into the lower part of layer 7 (US7den). This tunnel crosses horizontally the Mousterian stratigraphic sequence and without crossing the layer 5top and the layers above it.

The faunal remains of the Mousterian sequence (number of bones: 4.126 and NISP 171) record the predominance of carnivores (brown bears, cave bears, mustelids and rare wolf) over ungulates. Amongst the latter, the most abundant species are the cervids (red deer, *Cervus elaphus*, roe deer *Capreolus capreolus* and elk, *Alces alces*), followed by the caprids (chamois and ibex), and wild boar, *Sus scrofa*, and bovids (Bos vel Bison). Human interest in ungulates is evidenced by cut marks on red deer bones. Also, the remains of *Ursus spelaeus* and *Ursus* sp. from layers 7 and 5top show traces of butchering, skinning and the deliberate fracturing of long bones [35], [37].

The degree of fragmentation of the bones is elevated, as a result of diverse post-depositional processes and the activities of both carnivores and man. The remains bear alterations such as root micro-dissolution grooves, chaotic trampling, manganese coatings and concretions which can obstacle the recognition of the traces.

On the other hand, the taphonomic difference between the Gravettian levels is notable, because in these, the faunal remains present a more intense weathering, including exfoliation and extensive concretions, causing a marked yellowish colored surface (10YR8/3). Evidence ascribable to gnawing rodents is almost absent (NR 2), whereas small-sized carnivores seem to have been active in more recent times, seen predominately on the diaphyseal or epiphyseal remains of marmots (RN 19, with presence of pits and/or scores). Bird remains are rare, they come from layers 5top (1), 5 (11), 8 (2) and 10 (1), but appear well conserved. They show the same light yellowish color, four are punctuated of manganese stains, only two show the epiphyses alterated and one has been affected from microdissolution root alteration. No anthropic traces have been observed. In addition to the claw, these include the appendicular elements (five pedal phalanges and four claws) of birds of small to medium size, as well as a single sternum element (semi-intact sternum), a femur, and four indeterminate shaft remains of small sized birds.

The pedal phalanx belongs to the third left digit. It is complete and has punctiform concretions of manganese oxides, characteristic of the Mousterian bone material. It shows a fracture caused by dehydration that obliquely crosses the surface of the articular facet. The anthropic traces are short and are localised along the perimeter of the proximal articular facet (Figure 4, 1–2–3–5–6). In three cases (Figure 4, 1–5–6) the cutmarks suggest the cutting of the medial and lateral extensor tendons and ligaments. A localized stria on the upper margin of the articular facet (Figure 4, 2), in proximity to the dorsal proximal end of the keratin case, can testify to an incision of the cortical organic tissues or the tendon of the *extensor digitorum longus*. On the opposing margin, partial damage to the cortical bone tissue is clearly visible, in association with some microstriae (Figure 4, 3), probably created by flexing of the digit in a direction opposite to its natural articulation. Shallow sub-parallel scrape marks (Figure 4.4) are visible at the bony base of the talon where it articulates with the tendon *flexor profundus.* This incision, the only one with no transverse orientation to the major axis of the element, could indicate a clean removal of tissue or even of the same tendon, which occurred after the disarticulation.

### Mandrin cave

Grotte Mandrin is a vaulted rock shelter directly overhanging the Middle Rhône valley. The river reaches the shore of the Mediterranean coast some 120 km due south of the site. Research conducted since 1991 has enabled surface excavation of most of the vaulted area. Six stratigraphic units (A to F) have been excavated, yielding seven archaeological layers divided into five cultural phases, from base to top: 1) Quina Mousterian, 2) Neronian, 3) Post-Neronian I, 4) Post-Neronian II and 5) Protoaurignacian (Figure S2) [38]–[41]. These phases cover the final Middle Palaeolithic from 52/56 ka cal BP up to the early Protoaurignacian around 42 ka cal BP.

Mandrin Cave contains a reference Middle Palaeolithic to Upper Palaeolithic sequence, because it records all of the cultural phases currently known in Mediterranean France for the last Neanderthals up to the earliest Upper Palaeolithic. Each archaeological unit has yielded a rich lithic industry associated with diverse faunal material. Geoarchaeological investigation shows that the overall preservation state of the sedimentary context is from good (units A to D) to excellent (units E–F) and that a significant portion of the deposit was formed by windblown sedimentation of local sand and silts. Paleontological remains are exclusively related to the human activities in the cave, as shown by their high frequency of anthropogenic modifications (cut-marked bones, impact and notches on fresh bones, green fractures, burned bones, retouchers). Layers B to E have yielded several anthropogenic structures. The claw was found in layer E, belonging to the Neronian culture (Figure S3).

Excavation of this layer started in 2002 and has been excavated over an approximately 50 sqm area. This stratigraphic unit is one of the best preserved of the entire sequence. Archaeological field operations, as well as spatial and micromorphological analyses show that the spatial distribution of the remains is poorly affected by post-depositional processes [42]. This layer is also the richest archaeological unit of the excavated sequence. The faunal record is particularly diverse, indicating the hominin exploitation of a large spectrum of herbivores, and episodically wolves (whose bone remains are burned and exhibit cut-marks) as well as small game such as rabbit (*Lepus*), which also exhibit anthropogenic traces. The faunal assemblage is dominated by horse (*Equus caballus*) and red deer (*Cervus elaphus*) followed by steppe bison (*Bison priscus*) and ibex (Capra ibex); we can notice few occurrences of other cervids (*Capreolus, Rangifer, Megaloceros, Dama*), equids (*Hydruntinus*) and chamois (*Rupicapra*). Identified fauna among the scarce carnivore record includes predominately red fox (*Vulpes*), as well as few wolf (*Canis*) and very few bear (*Ursos arctos*) and mustelid (*Meles*, aff. *Martes*) remains [42]. As seen across the sequence, the herbivore remains are mainly related to human subsistence activity. Lithic end-products –i.e. domestic tools and weapons-are numerous, and the highest representation of points ever recorded in the European context, with more than 800 points, commonly of microlithic size, of a specific industry attributed to the Neronian culture [38]–[40].

Layer E yielded 11 bird bones belonging to Galliformes (Capercaillie *Tetrao urogallus*, Gray Partidge *Perdix perdix* and Common Quail *Coturnix corturnix*), Passeriformes (Alpine chough cf. *Pyrrhocorax graculus* and undetermined taxa) and Accipitriformes orders. This last taxon is represented by a single bone, a terminal pedal phalanx of Golden eagle *Aquila chrysaetos*). This bone is the only one that shows clear evidence of human modification. Other marks on the bird remains indicate intervention by non-human predators: two Passerifomes bones carried light digestion marks, probably made by a nocturnal raptor, and an undetermined Aves fragment is modified by carnivore teeth.

Chronology of layer E is based on a large corpus of TL and ultrafiltrated AMS C14 dates, enabling the creation of a Bayesian model out of some 30 radiometric dates. The model suggests that the Neronian dates are grouped between 52,480–48,130 cal BP (68.2% prob.) and 55,260–46,620 cal BP (95.4%). A cautious interpretation is that Level E dates to ∼50,000 cal BP with an uncertainty of 3–4000 years, but is likely to be older rather than younger [42].

The terminal pedal phalanx (talon), which belongs to a right digit 1 of a Golden Eagle, shows several cut-marks. All are located on the proximal end of this well-preserved bone. A deep mark, partially filled by manganese, is visible on the medio-anterior face (Figure 5, 1). Two other parallel cuts are observable on the protuberance of the talon on the latero-plantar face (Figure 5, 2). The similar morphology and orientation of these two marks suggest that a single cutting edge was used in a repeated movement. A last deep striation is located on the center of the protuberance of the talon (Figure 5, 3). The orientation and locations of the three groups of cuts indicate that craftsman have used several gestures in order to detach the talon. An unretouched-flint edge was probably used, as suggested by the morphology of the most well-preserved marks (Figure 5, 2–3). In the current state of research, this piece represents the first clear evidence of bird exploitation from the Neronian tradition.

## Discussion and Conclusion

The discovery of two further raptor pedal phalanges in addition to those already known in the Middle Palaeolithic of Europe stimulates discussion on various aspects of Neanderthal behavior related to the acquisition, processing and the intended use of these avian elements.

Few contributions now attest that the predation of birds was practiced since the Early Middle Pleistocene and that this predation was directed towards edible species, with clear evidence of consumption in Mediterranean Europe in Gran Dolina cave TD10 and Bolomor cave layers XVII, XII, XI, IV [43]–[46]. However, hunting and gathering or the processing of raptors stays in question. In Rio Secco and Mandrin caves, as at Pech-de-l’Azé I and IV, Combe-Grenal, Les Fieux and Fumane Cave, the large raptors and the swan from Baume de Gigny are only attested by single phalanges, which do not enable a global patterning of raptor exploitation. Such contextual evidence available for the chronological period provides scanty data, and in all cases remains less represented than the claws. In fact, although the raptor bone remains are common within Mousterian levels [1], evidence for the exploitation of these birds is scarce. Vanguard Cave in Gibraltar yielded a cut-marked griffon ulna [1] and Morin and Laroulandie [9] have reported traces ascribable to the defleshing or disarticulation of a Golden eagle proximal femur and an indeterminate falcon distal humerus respectively from Les Fieux (level K, denticulate, sector west) and Grotte du Noisetier, France, which at a general level cannot exclude the occasional consumption of raptors by archaic humans.

Because claws are not edible or consumable material, the Rio Secco, Mandrin and other specimens do not meet the nutritional requirements for human subsistence. Their recovery can be done on a dead individual, as bird feet represent a fleshless part of carcass that can dry naturally. In such a case, the scaly skin preserves the bones and phalanges, which stay in connection for a long time after the complete decomposition of fleshy parts. However, the recuperation of the talon required a tool, and this task was carried out in a precise manner, specifically aimed to not damage the corneum. As observed in the experiment, the extraction can be performed in two modes, dorsal and/or plantar, sometimes even assisted by manual flexion. In both cases, the resistance of the lateral ligaments requires an energetic intervention and the use of a lithic tool that avoids damage to the extremity of the keratin case. In fact, it was found that flexion, especially if performed on the plantar surface, can damage the ends of the keratin case. Indeed, the keratin structure bends exactly near the end of the internal bony claw, thus losing its original stiffness and becoming more vulnerable to mechanical stress.

The experiment therefore suggests that all operations could be aimed toward the detachment of the intact phalanx without damaging it, but it could also not be excluded that the tendon was among the objectives of the intervention. In fact, dorsal disarticulation permits the recovery of the claws with the *tendon flexor profoundus* still connected. However, when this is removed or cut, additional as well as multiple incisions are produced, exactly comparable to those conserved at the base of the bony claws of Rio Secco and Mandrin.

Overall, it could be suggested that the deliberate removal of the claw can be ascribed to a functional purpose. However this is only partly determinable, because, if the functional purpose involved the use of the pointed claw, for example, in drilling, its perishable composition does not allow the preservation of any scars or polishes imprinted on its surface. In addition, the fragility of the acute extremity and its poor resistance to wear make it a less suitable and durable tool. Further, one could assume the use of the bone element, which is as yet unattested, even in the Upper Palaeolithic and Mesolithic. Rather, evidence relating to these periods has been interpreted as likely items of symbolic expression [13].

A particularly noticeable clue is that all of the cut-marked claws found in Middle Palaeolithic sites come from eagles, which argues against their utilization in strictly non-symbolic contexts. Its use as an ornamental element could take different forms that are still difficult to detect due to the disappearance of the corneum case or the lack of diagnostic wear or even traces of ocher or other coloring materials on the surface. It has been suggested that suspension of the element was possible with a string tied between the base of the heel and the articular facets, but the absence of diagnostic wear on all specimens analyzed refutes this hypothesis. A variation would imply a decision not to cut the 10 cm long *tendon flexor profoundus* in order to suspend the same claws, but the presence of cut-marks at Rio Secco and Mandrin also further argues against this possibility.

**Figure 6:**
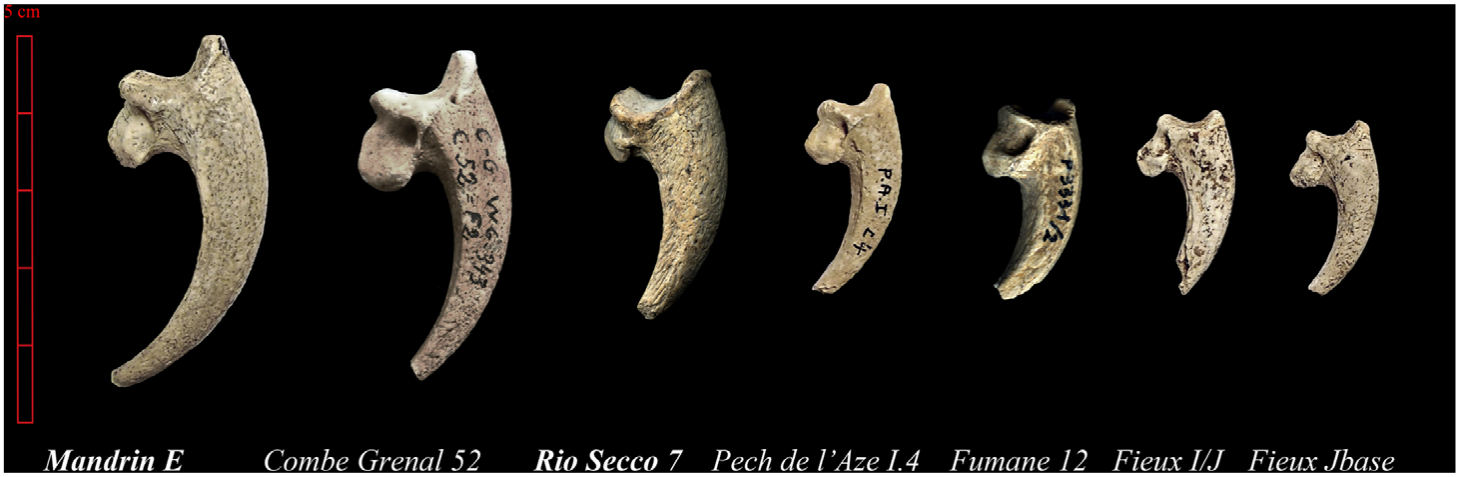
Cut-marked claws from the European Middle Palaeolithic. Presentation of the cut-marked eagle bones found in the Mousterian sites cited in the text and ordered according to the size.

**Figure 7:**
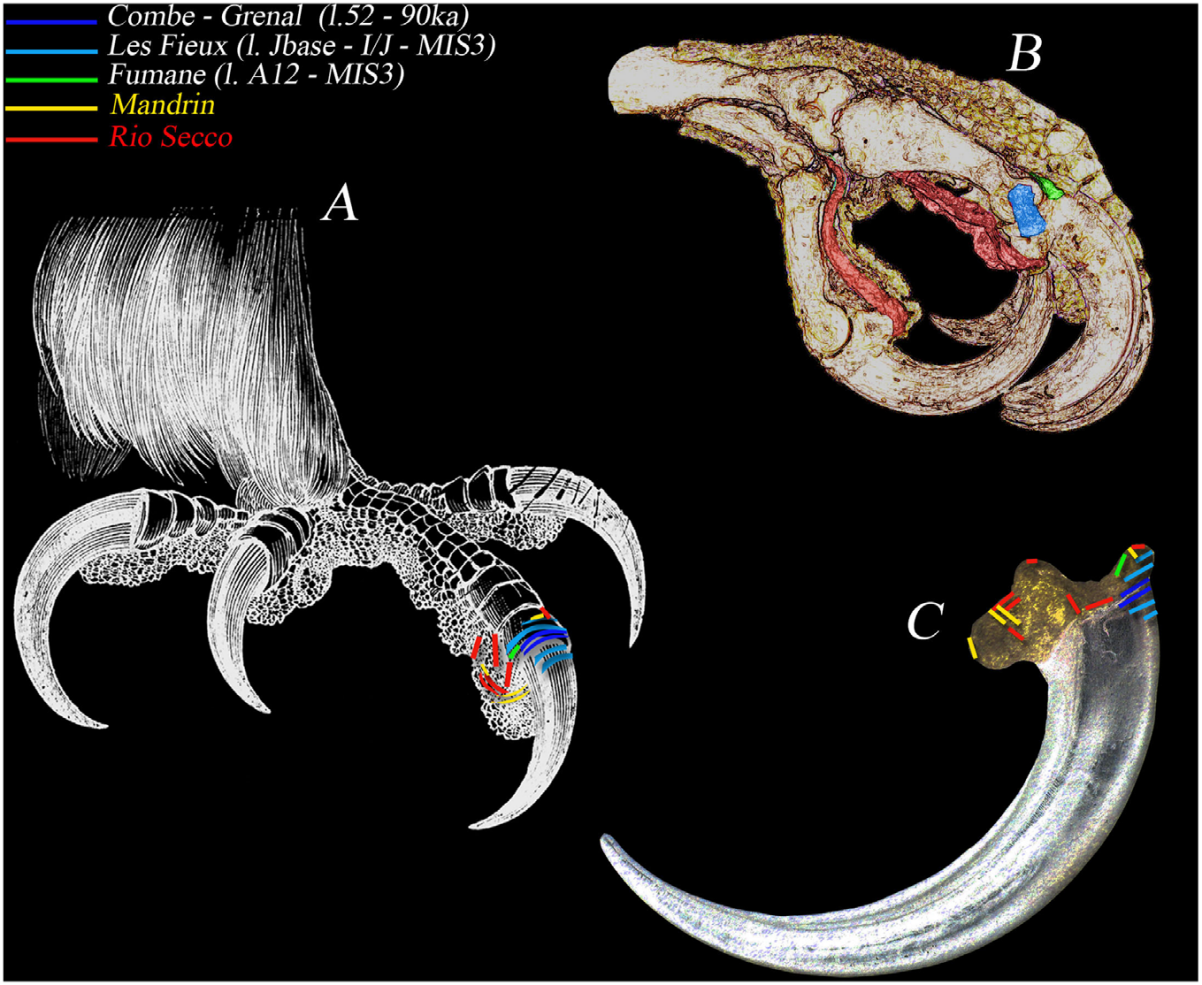
Summary of the cut marks observed on the Golden Eagle left foot. A) the left foot with the summary of the cut marks known in the European Mousterian sites above the organic tissues (keratin case, dorsal thick cotica (B) and terminal keratineous fingertips). B) section of the Golden Eagle left foot with evidence of the strong tendons: green = *tendon extensor, general and own;* blue = *medial and lateral tendons extensor*; red = *tendon flexor profound* (length of 10 cm). C) articulation of the bony core of claw and keratineous case with localization and orientation of the all traces ever recognized at the time being. The colors are related to the different sites where similar traces are present in the same anatomical elements (see figure legend).

A comparison between the pedal phalanges found in the other Mousterian contexts across Europe shows differences in the pattern of the incisions (Figures 6 and 7). At Pech de L’Azé I, level 4, two cut phalanges (1 and 3) and a claw from the digit III were the only remains of the Golden Eagle [4]–[5]. The talon does not show marks but striae on the dorsal face of the penultimate phalanx indicate detachment of the claw. At Combe-Grenal layer 52 (≈90 ka), the golden eagle terminal phalanx bears on its proximal-dorsal side two incisions that closely coincide with the proximal margin of the keratinous sheath overlying the terminal phalanx of the digit. At Les Fieux (≈45–50 ka) the two white-tailed eagle terminal phalanges, one from stratigraphic unit Jbase that is associated with the Mousterian of Acheulean Tradition industries and the other from the unit I/J, which is associated with the Denticulate Mousterian, exhibit cutmarks in a similar anatomical location. Striae on the talon of a large diurnal raptor from the Ahmarian layer B of Uçağızlı Cave (≈34 ka) [14], and one *Bubo* sp. claw from la Quina [11] are also at the same location. At Fumane, layer 12 (>46.4 ka), the golden eagle bone associated with a Levallois Mousterian industry [47], [10] shows one single trace that was certainly made when the phalanx was already exposed. The position on the articulation, below the protruding upper dorsal ‘beak,’ is transversally oriented to the main axis of the element, and the absence of dorsal striae, such as those seen at Combe Grenal or Le Fieux, is attributable to both a plantar and dorsal incision, in a more proximal position in respect to the articulation, so as to exclude the edge of the keratin case. At Pech de l’Aze IV, layer 8 (≈100 ka), the talon of a medium-sized raptor exhibit a cut-marks at the same location as seen at Fumane [7]–[8]. Few Snowy owl claws from upper Magdalenian (≈15 ka) sites in south-west France show disarticulation marks at this same location even if the main pattern of marks is on the plantar protuberance [16]–[17].

Two main ways for removing the claws can be envisaged in the archaeological record. Nevertheless, these do not seem to reveal strong cultural significance. On the contrary, the few cases mentioned above seem show a convergence, in that the constrained anatomy of this part of the avian body may influence decisions and handlings.

Eagles and large raptors in general are among the rarest birds in nature, due to their top position in the trophic chain, and this may indicate that these large and powerful diurnal birds attracted hominins and stimulated their use as a symbolic media by Neanderthals, joining a universal trait of sub-actual societies [1]. In that way, eagle claw selection may well build a bridge between sapiens and Neanderthal behaviours, suggesting some shared cognitive traits amongst these populations. At the end of the Middle Palaeolithic, the use of raptor terminal phalanges (Rio Secco, Fumane, Mandrin, Pech de l’Azé I, Fieux) may be interpreted as a wide circulation of ideas potentially built through symbolic mediations and underlying a much more complex social network than expected. Finally, our approach proposes some gradation in the use of symbolic demonstrations. A major advance would be now to demonstrate positively that symbolic materials have existed among these societies, through the discovery of unambiguous modifications of objects in order to ornate them or to create a proper morphology for their suspension. Such positive evidence would shed a heuristic light on these too exceptional finds of negative evidence. One may argue that in later contexts, like during Upper Palaeolithic or Neolithic, such elements are interpreted as pendants and ornaments, but these elements make sense only as these later contexts previously provided thousands of various categories of positive evidences. It is undisputable that concerning *Homo sapiens* and following Abraham Maslows pyramid of human needs, human behaviour is “usually meaningfully linked to temporal, spatial and social configurations” [48]–[49]. But going past and crossing other humanities, the main ambush would be a crude projection of our own needs on fossil hominids for whom behaviours still need to be precisely and guardedly evaluated. Since then, Neanderthal behaviour retains a part of its mysteries, a complexity that should not be reduced to the specific behavioural sphere of *Homo sapiens*.

## Materials and Methods

Ethics Statement. All necessary permits were obtained from the Italian Ministery of Culture and the French Ministery of Culture for the described study, which complied with all relevant regulations.

The birds exploited for experimental butchering were carcasses naturally or accidentally died and previously stored in refrigerators at, respectively: the Reproduction and Rehabilitation Center of Raptors at Cornino, Folgaria, Italy (griffon vulture and eagle-owl), and the Center for Protection of Owls and Raptors, Haringsee, Austria (bearded vulture). All permission for using these carcasses where provided by these two centers.

The unique identification number of the specimen analysed is RA23026 for Rio Secco Cave and MAN05 E 474 for Mandrin Cave.

The bones and the experimental unguals phalanges (cleaned from organics tissues with water) have been examined at low magnification (10–20X), and with stereomicroscope Leica S6D Verde Ough (0.75–70X) at the Archeozoology and Taphonomy Laboratory, University of Ferrara (LAT). Claw from Mandrin was observed under a Microscope Leica Z16APO at the University of Bordeaux.

Repository information: the Fumane specimen is temporary housed at the University of Ferrara, in the Section of Prehistory and Anthropology, Ferrara, Italy, with the permission of the Ministry of Culture–Friuli-Venezia Giulia Archaeological Superintendence; the Mandrin specimen is temporary housed at the University of Bordeaux, in the Section of Prehistory and Anthropology, Talence, France.

The archaeological deposits at Rio Secco were systematically excavated within 50 cm subsquares. All ≥5 cm complete or fragmented lithics, bones, teeth and identifiable faunal fragments of ≤5 cm were 3D plotted. Smaller remains were recovered from 2×2 mm wet sieving and attributed subsquares and sub-units. Bird remains from layers 5top, 7, 5 and 8 are still in course of analysis at the anatomical and taxonomical level, while qualitative taphonomic modifications were recorded.

## Supporting Information

Figure S1 **Sagittal (A) and transverse (B) stratigraphic sections at the Rio Secco cave, stratigrapic log (C), view of the upper boundary of the Middle Paleolithic layers with position of the finding of the claw (D, yellow).**
(TIF)

Figure S2 **Sagittal stratigraphic section of Grotte Mandrin.**
(TIF)

Figure S3 **The discovery of the bone claw at Grotte Mandrin.**
(TIF)
