# Supplementary figures and images for: Convergent Evidence of Eagle Talons Used by Late Neanderthals in Europe: A Further Assessment on Symbolism
Source: PLoS One. 2014 Jul 10;9(7):e101278. doi: 10.1371/journal.pone.0101278 (PMC4092065; doi:10.1371/journal.pone.0101278)

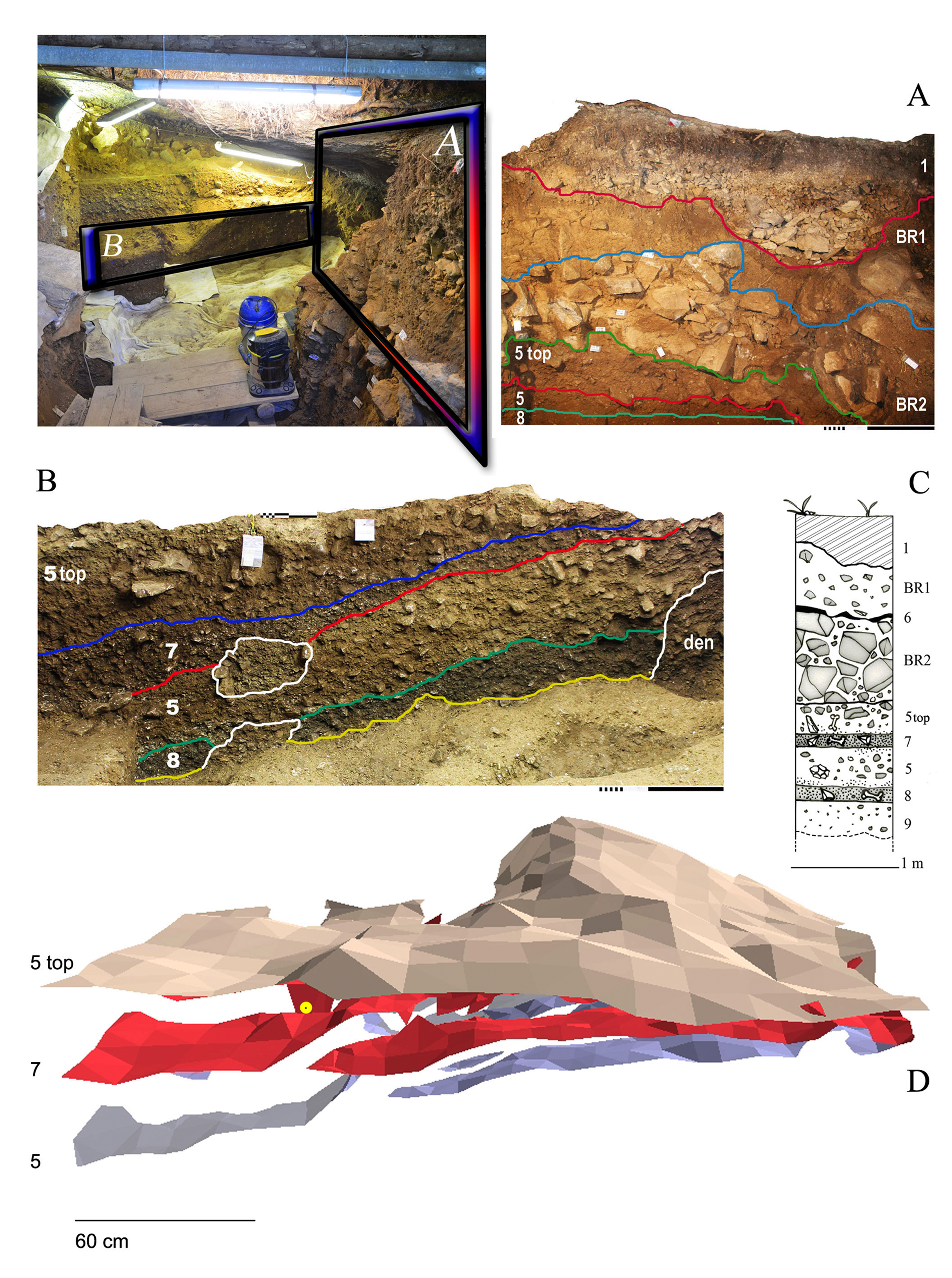

Supplement: Figure S1 — Sagittal (A) and transverse (B) stratigraphic sections at the Rio Secco cave, stratigrapic log (C), view of the upper boundary of the Middle Paleolithic layers with position of the finding of the claw (D, yellow). (TIF) [file pone.0101278.s001.tif]

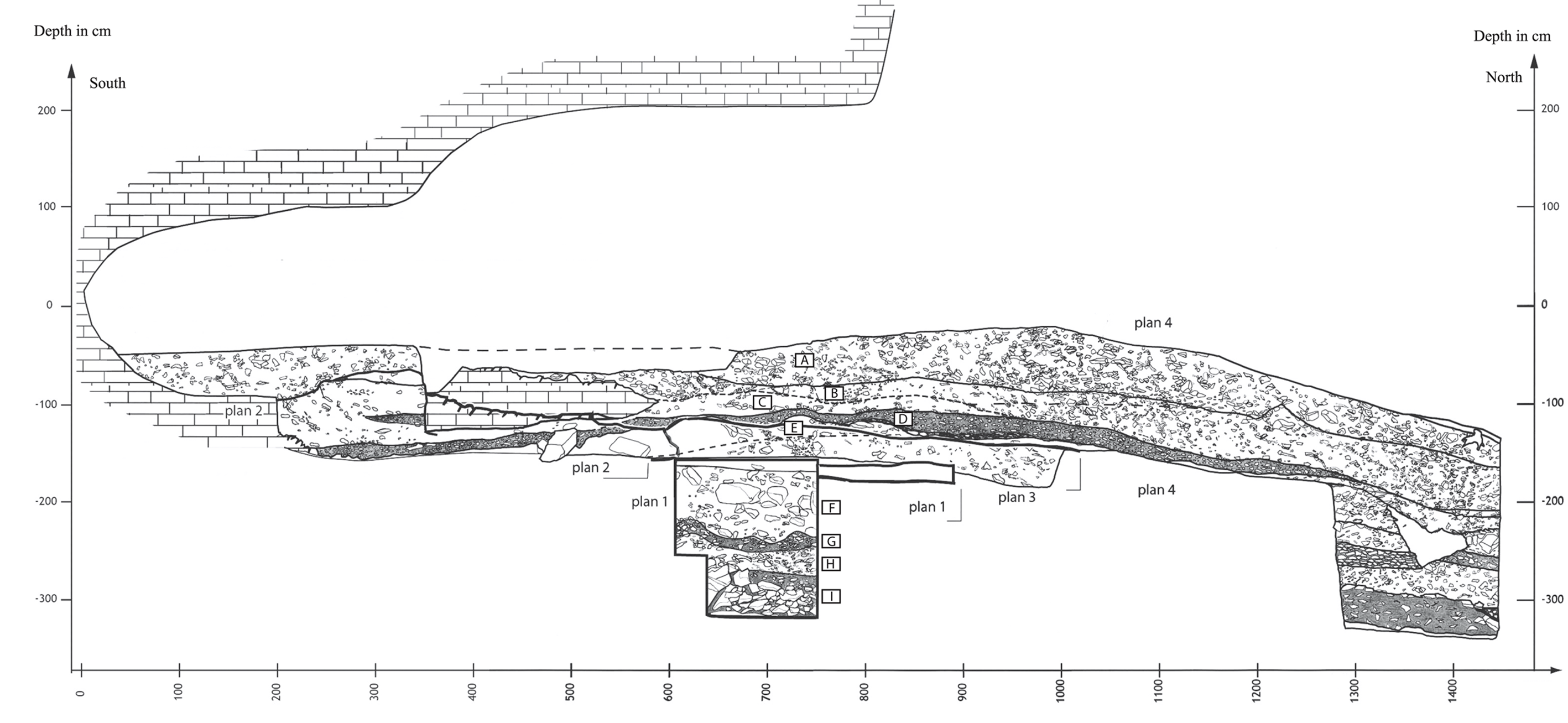

Supplement: Figure S2 — Sagittal stratigraphic section of Grotte Mandrin. (TIF) [file pone.0101278.s002.tif]

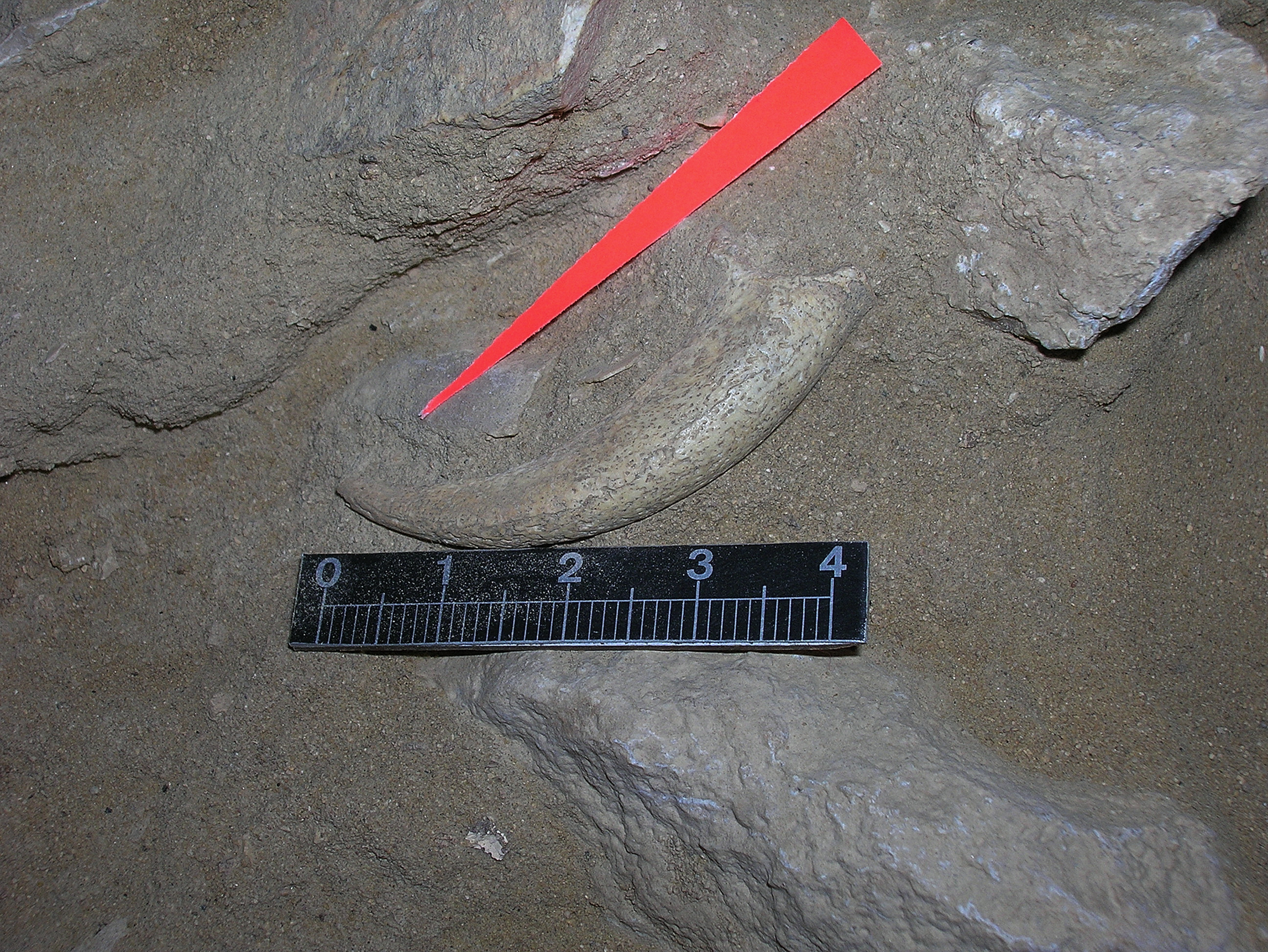

Supplement: Figure S3 — The discovery of the bone claw at Grotte Mandrin. (TIF) [file pone.0101278.s003.tif]
